# Inclusive capitalism: The emergence of a new purpose paradigm in economics and business administration and its implications for public policy

**DOI:** 10.1007/s43508-021-00020-z

**Published:** 2021-07-07

**Authors:** Martin de Jong

**Affiliations:** 1 Rotterdam School of Management & Erasmus School of Law, Erasmus University Rotterdam, Rotterdam, The Netherlands; 2 Institute for Global Public Policy, Fudan University, Shanghai, China

**Keywords:** Inclusive capitalism, Capitalism, Inclusion, Purpose paradigm, Economics, Business administration, Public policy

## Abstract

With the societal cracks resulting from decade-long neoliberal policies becoming increasingly visible in many countries, capitalism as the most suitable institutional system to produce material wealth, environmental sustainability and social stability has come under growing attack. This contribution examines what the growing army of recent heterodox scholars in economics and business have to say on what one could call ‘inclusive capitalism’. This concerns both the flaws in current capitalist systems and the behavioral assumptions that underpin it, as well as the possible institutional fixes they propose. I first sketch the background of the crisis surrounding capitalism, delve into its conceptual foundations and offer a working definition. I subsequently examine what social and environmental inclusion refer to and use Kate Raworth’s conceptualization of the doughnut economy as a point of departure to explore what ‘inclusive capitalism’ may imply. I also identify requirements for its implementation in institutional practices. It appears that ‘purpose’ rather than utility maximization or profit maximization is what novel economists and business scholars perceive as the key driver in ‘stakeholder-oriented capitalism’ or the ‘economics of mutuality’. Their claim is that at the end of the day this is not only a moral imperative for companies but also more beneficial for them in terms of long-term profitability. Moreover, they see a far more important role for government in shaping markets and leading the way into a more inclusive future than it is currently fulfilling. I argue that it is time for scholars in the field of public policy to take heed of these new theoretical developments in neighboring disciplines and respond to them.

## Introduction

While most scholars in the field of public policy are happily engaged in working out further details of normal theory in New Public Management and New Public Governance, in several other social science disciplines, a paradigm shift seems to be under preparation. Not only in sociology or geography, but also increasingly in economics or business administration, neoliberalism has become a swearword and modern-day capitalism is either rejected or at least deemed in urgent need of repair (Steger & Roy, 2021). Milton Friedman and his ideas on policies to control the flow of money, his moral yardstick for companies only to maximize shareholder value and his methodological treatise about economics being a science of prediction rather than description and explanation, not unlike handling billiard balls that simply have to run their course to win a game, have been fashionable for decades. Economists leaned on his maxims for modeling and forecasting. Business scholars and organization theorists mimicked his idea that ‘the only business of business is business’ and corporations were in the end mere money-making machines. Political and policy scholars joined the fashion and proposed to shrink government and limit the damage it did to society, while the remainder of social science mapped societal problems maintaining a firm belief that these were only minor ripples in the river; and that overall humanity was on a speed-boat to inevitable progress. Left-leaning geographers and sociologists who offered systematic criticism could easily be pushed aside as ‘radicals’ or ‘Marxists’.

But the tide is turning. The past decade has seen a growing number of influential scholars from leading universities disavowing the mainstream assumptions underlying the academic disciplines they grew up in. Kate Raworth (2017) and Mariana Mazzucato (2018) laid bare the serious limitations of modeling exercises based on erroneous assumptions and demonstrated how *ceteris paribus* clauses were abused to favor hobby horses over societal relevance. Paul Collier (2018) and Jonathan Aldred (2019) showed how these assumptions were not just erroneous and based on an overly negative conception of human nature, but also how they lead to policies rewarding selfishness as dominant patterns of behavior and systematically corrupt our morality. Colin Mayer (2018) and Rebecca Henderson (2020) have spotted how maximizing shareholder value has led companies to sacrifice long-term investment and employee income for the benefit of self-enriching owners and top managers, while mostly neglecting the primary societal goals these companies were aimed to fulfill at the outset. Joel Bakan (2004, 2020) and William Lazonick and Jang-Sup Shin (2020) essentially characterize the modern corporation as a predator with the greediest and most temporary shareholders killing ‘their’ corporations the quickest by pulling out massive financial and other resources and lobbying government with enormous funds to further financialize the economy and deregulate any control the public sector might have over their discretionary operations. Meanwhile, the major environmental challenges and issues in decreasing social stability are left for a deeply battered and torn government to resolve.

However, if the Washington consensus is dead even in Washington itself and neoliberalism is not even defended anymore by the political right, what will come in its place? Since acquiring and accumulating capital is still the guiding behavior, and an essential basis of existence for many of us, we must assume that capitalism is so deeply ingrained in our organized systems of production and consumption that it will not disappear any time soon. But then, how can or should it be reformed? Thus far, a variety of the abovementioned academic disciplines have produced intriguing first building blocks to scaffold such a reform and displace old mainstream elites in their profession from their positions of power. The political and policy sciences, however, have been remarkably absent and appear to be latecomers in the debate on reforming capitalism. Although it is understandable that policy scientists shun impactful and potentially explosive ideological terminology and to some extent reflect the grayness of the public bureaucracies they study, there is no way around it this time. If socio-economic and environmental inclusion are to take shape in ‘New Green Deals’, if weakened public organizations should no longer be meekly following and pleasing emboldened private firms, but check on them, lead them and guide them towards a more equal and sustainable society, they should be readied for that new position. It falls partly on scholars in politics and policy to offer them conceptual ammunition for this transformation, invigorate their own discipline to perform this task and converse with economists and business scholars on equal terms. In this contribution, I will attempt to take a first step in this direction and synthesize the lines of thought as they have been developed in the new ‘purpose paradigm’ as it is emerging in economics and business administration. I will focus primarily on these two disciplines with occasional sidesteps to economic sociology and economic methodology, but not touch on other areas. Neither will I claim completeness in my presentation of these influential fields or assume that all authors I mention agree with each other on all aspects of their take on capitalism and the direction of its reform. What I do intend to do is present a stylized version of what ‘capitalism’ currently is, what crucial weaknesses it has and in which direction the new ‘purpose paradigm’ claims the institutions undergirding it should be transformed to make it more ‘inclusive’.

The remainder of this article will proceed as follows. Section 2 will examine what can be considered the key features of ‘capitalism’, which of them can be seen as pernicious for generating an inclusive society, which appear unalterable given the path-dependency of socio-economic evolution and which institutions can and should be reformed. In Sect. 3, the concept of inclusion will be central. I will investigate its elusiveness, which main environmental and social dimensions it covers and how different dimensions of inclusion may go against each other and necessitate trade-offs. Section 4 will then be introducing ‘purpose’ rather than ‘utility maximization’ or ‘profit maximization’ as the main driver underlying human and corporate behavior and require a stakeholder orientation rather than a shareholder orientation within firms to realize inclusive capitalism. In Sect. 5, institutional differences across ‘varieties of capitalism’ around the world will be central, along with the question of how they affect the inclusiveness of their system. Explicit recognition of the value of other types of capital beyond finance alone can take them a long way on their transformation to higher levels of environmental and social inclusion. Finally, in Sect. 6 I will present the main conclusions of my inventory of this new ‘purpose paradigm’ and the implications this body of literature for the future development of public policy as a discipline.

## Defining capitalism without ideological presuppositions

It is not unusual for analysts to use the term capitalism and give it obvious positive or negative connotations. Neo-institutional economists such as Oliver Williamson or liberal sociologists like Daniel Bell may speak of the ‘economic institutions of capitalism’ and certain limitations these might have, but they would not for the world want to give them up for fear of opening the floor to non-democratic alternatives (Bell 1976; Williamson 1990). Neither would business analysts Bruno Roche and Jay Jakub (2017) when they explain how corporations can improve their supply chain and enhance their profitability by redirecting their production processes in ways that enhance rather than degrade the human and physical capital of the partners they work with: they call that ‘perfecting capitalism’. And as optimistic believers in a bright future beyond capitalism are hard to find, it takes pessimists such conservative urban scholar Joel Kotkin (2014, 2020) or neo-Marxist sociologist Wolfgang Streeck (2016) to predict the coming of a neo-feudal society emerging from the ashes of dramatically grown policy quandaries to which governments are no longer able to face up and dramatically grown socio-economic inequalities which can easily be called a societal structure based on new class distinctions. If the optimism of a new communist hosannah is understandably shared by very few, it appears that post-capitalism is rather a synonym for an undefined, grim and inequitable complex of human institutions and constellations that is to be feared but cannot be avoided.

And yet, before jumping to conclusions, it is important to make an attempt at impartiality when characterizing the socio-economic institutions that define capitalism. Thomas Piketty (2017) and Katharina Pistor (2019) lead the way here by harking back at *the* essential component of capitalism: capital. Once humans invented and deployed currency, this allowed for the use of metal coins not only as a unity convenient exchange and calculation, but also as one of storage. This latter proved especially consequential because it lies at the origin of the drive to collect, invest and collect sometimes unfathomable amounts of money. Once early technological development made the production of agricultural surpluses possible, trade grew and accumulation of capital among merchants became possible, the relative importance of business ventures with invested sums started on a steep incline. Where and when ‘capitalism’ officially originated remains a matter of dispute, but there is little disagreement that international explorations and exploitations triggered technological progress and societal upheaval with the accumulation of capital evolving into a key driver underlying the behavior of at least the financially successful segments of humanity. This socio-economic transformation led to modes of production and consumption where the relative positions of the various production factors changed position vis-à-vis each other. Feudal societies in which agriculture, the primary economic sector, was the dominant economic function consisted largely of an interplay between land (nature) and labor, with the landlords as owners of the land controlling and squeezing resources from the owners of labor. The activation of capital as a meaningful additional production factor evolved into a tipping point for societal change: while self-sufficient supply of agricultural produce allowed cities and factories to grow, the relative importance of land decreased and it was the relationship between the owners of capital and those of labor that defined production and consumption processes in society: while the relations between landlords and serfs constituted the main axis of interaction in feudal society, the interaction between capitalists and laborers is key in capitalism.

Capitalism, however, is not a stable or stagnant mode of production. It has gone through various stages: global expansion has deeply affected it and the mediating role of governments has changed over time under the influence of power struggles waged by the main political players (Fulcher, 2015). Equally importantly, in recent decades the scarcity of land (nature) has grown under the influence of intensive use of natural resources, the abundance of labor has risen due to dramatic population growth, the availability of financial capital has become less problematic than it was before and the impact of a fourth production factor, knowledge and data, on the other three and its comparative value has grown enormously, leading many to believe we have entered the era of a knowledge economy. Farms and factories continue to produce, but they can now be found side by side with offices and laboratories in which the processing of data is the main activity. In other words, the labor input and contribution to Gross Domestic Product of the primary sector (agriculture) has dwindled but it is not negligible, that of the secondary economic sector (manufacturing and mining) was at its zenith a few decades ago, but is now falling rapidly in most parts of the world, while that of the tertiary sector (services) has been augmented accordingly. This transformation of the industrial structures over time has also altered the strata or class-divisions in society. Although there are still remains of the landlords, yeomen and day laborers in what is left of the primary sector and capital owners, small manufacturing firms and workers still exist, they have been supplemented by what Richard Florida (2003, 2018) calls a creative class of professionals and artists and a service class of lowly skilled, lowly paid and temporary employees with high levels of vulnerability at the labor market. In sum, class structures in twenty-first century capitalism differ markedly from those in the nineteenth century, but they have anything but vanished. Joel Kotkin’s (2020) idea that modern neo-feudal society now consists of an oligarchy (similar to the feudal nobility), a clerisy (analogous to the feudal clergy), yeomen (independent owners of small and medium enterprises) and serfs (members of the working and service classes) may be oversimplified, but ultimately not so farfetched.

However much capitalism may have changed over the decades and centuries, according to leading theorists it does have a number of features that really define it as such. It is economic methodologist Geoffrey Hodgson (2015) that has made a comprehensive historical and conceptual study of its essential and indispensable features. In his summary definition, which I fully embrace here, capitalism has six main characteristics (Hodgson, 2015: 259):

1. A legal system supporting widespread individual rights and liberties to own, buy and sell private property,
2. Widespread commodity exchange and markets involving money,
3. Widespread private ownership of the means of production by firms producing goods or services for sale in the pursuit of profit,
4. Much of production organized separately and apart from the home and family,
5. Widespread labor and employment contracts,
6. A developed financial system with banking institutions, the widespread use of credit with property as collateral, and the selling of debt.

In my view, none of these features are likely to disappear any time soon, leading to the conclusion that any form of environmentally and socially inclusive economic system will have institutions that change political, legal, organizational or cultural patterns without touching on these six aspects. Individual political and legal liberties, markets, private ownership, labor done for organizations based on employment contracts and capital transactions through financial institutions are unlikely to be disbanded, so what is left?

## Defining inclusion without ideological presuppositions

Arguments that it is vital to realize inclusion and inclusiveness in society have grown considerably more powerful and prominent in recent years. However, as with many popular container concepts, this obviously means different things to different people. Given the vastness of the literature on the topic and its dissemination far beyond the realm of economic systems, I will restrict myself to the literature on the institutional aspects relevant to managing capitalism. It is easiest to delineate the inner and outer reaches of inclusion with the help of Kate Raworth’s (2017) conception of the doughnut economy. She defines an outer boundary beyond which economic production, distribution and consumption cannot go: the ecological carrying capacity of planet Earth. This implies delving and digging natural resources only to the extent that these can be replenished, emission of harmful substances in air, water and land to levels that keep eco systems intact and eventually the complete closure of reduce-reuse-recycle flows to prevent water, energy and materials from becoming irreparable waste. One could call maintaining this ecological outer boundary or upper level realizing ‘*environmental inclusion’*. In many ways, implementing environmental inclusion coincides with what is known as the environmental part of the global sustainability agenda and what still others have called the circular economy. The academic sub-disciplines environmental economics (for weak sustainability) and ecological economics (for strong sustainability) have been established for academically rigorous thought on and policy measures promoting environmental inclusion when looking at them from an economic angle. There is a difficulty, however. The former converts any type of natural good or bad into utility or monetary terms and therefore simply reduces nature and its various elements to just their monetary and/or utilitarian value and does so with the help of primarily financial instruments. In contrast, the latter line of thought does honor the multi-dimensionality of various aspects of environmental degradation, but it can rarely present conclusive approaches to policy practice in which human and non-human interests are combined into convincing policy packages. Replacing coal with nuclear energy is good for reducing carbon dioxide emissions and preserving precious landscapes, but it otherwise generates intractable and dangerous waste streams and an infinitely small likelihood of an infinitely large explosion disaster. Building compact cities with a great many high rise buildings is good for controlling energy consumption and making public transport affordable, but it is a potential threat to local air quality, beneficial direct human access to greenery and it increases the impact of the heat island effect typical of built environments. It is key to understand nuance in these possible interventions to secure environmental inclusion, but the required trade-offs are political rather than economic. Environmental inclusion eventually boils down to making important but arbitrary trade-offs among various dimensions inherent in natural resources and the impact of their use on human prosperity.

This same multi-dimensionality exists in the socio-economic inner boundary distinguished by Raworth. In her view, there exist certain minimum levels for enjoying acceptable quality of life, for humans to spend decent and meaningful lives in their respective societies: so-called *socio-economic inclusion*. These conditions certainly include material living conditions, but they are also far more comprehensive than wealth and income alone. Health, housing and absence of discrimination are for instance also part of the equation here. As inclusionary claims these various facets of sufficient quality of life for all do not universally point in the same direction. Nilson Ariel Espino (2015) uses anthropological argumentation to clarify why groups and individuals in society are often eager to accentuate their distinctiveness from ‘lower’ others and undertake action and deploy rules to restrict rather than promote inclusion. Robin Hambleton (2014) makes an impactful plea for maximum levels of social, political and economic inclusion on all counts to generate societal fairness but has to acknowledge that realizing inclusive inclusion is in the end a truly tough game. The former emphasizes limited cultural acceptance of full inclusion, because the privileged see it as going against their interest to accept equality in many aspects of life: they prefer to dress differently or live in separate neighborhoods. The latter presupposes a moral imperative (and in its wake a political desirability) of maximum broad and deep societal inclusion to realize a democratically just society. Taking a pragmatic rather than skeptical or idealistic stance, Anttiroiko and de Jong (2020) formulate the creation of shared prosperity as their key objective in realizing social inclusion and have introduced a list of so-called *exclusion grounds*: age, mental & physical disability, religion & ideology, race & ethnicity, gender & sexuality, income & wealth and location. Inclusion is obviously, here as well, a monster with multiple heads. Exclusion from (or inclusion in) benefits of any form of capital (human, social, financial, physical or natural) can be based (and often does occur) on any of these grounds. Being a minor can be a reason not to be admitted to public buildings, not having sufficient salary or owning real estate as collateral can deprive people from access to financial loans and being homosexual may in some societies preclude people from adopting children. Such exclusion can clearly be enforced by legal means, but even without the existence of legal restrictions, de facto exclusion through intentional or unintentional discriminatory practices is also very common. Although in political and social debate, it is normally explicitly or implicitly assumed that for all individuals and groups to be included in as many facets of life as possible is a *conditio sine qua non* for an ethically responsible society and should be considered a political and legal entitlement, in practice it appears that it is frequently impossible if not even sometimes unwished to include all individuals or all groups in all types of events or benefits. Voting may not be wise at any age or at any level of mental ability, access to the swimming pool may not be granted to any age and gender at any time, nor can or should all organizational positions be open to any level of educational qualification and nor are all religious facilities comfortable with allowing all individuals of any faith to all of its spiritual services. Different nations, cities and organizations structure access to their respective types of capital in sometimes very different ways, but in none of them is access totally open for all to every benefit and neither is it easy to pinpoint which type of inclusionary priority setting is clearly superior to any other. In short, both for ethical and practical reasons inclusion based on trade-offs across various interests but aimed at economic value creation rather than the imposition of inalienable rights and entitlements, generates higher levels of shared prosperity in any given society. If this balancing out among different exclusion grounds and access to benefits is based on a vision adopted and promoted by relevant authorities and/or leadership, but based on broad stakeholder involvement, it is likely to lead to higher levels of active participation of various societal groups in economic value creation. This occurs by recognizing well-known and lesser known talents these groups have and the constructive development and use of which generates higher levels of inclusive prosperity.

## Purpose as the cornerstone of an inclusive capitalist system

The behavioral assumptions underlying ‘homo economicus’ have long been lambasted by many heterodox economists and scholars in other social science disciplines alike. In their latest work ‘Greed is Dead’, Paul Collier and John Kay (2020) state the following about him/it:

The practical workhorse of market fundamentalism was Economic Man, an unappealing mammal who responded only to financial incentives. Greedy, selfish and potentially lazy, he exemplified *possessive individualism*. And smart: he knew all that was knowable. In the key phrase used by some economists, he ‘knew the model’ that described how the world worked. And the model showed that individual greed could be harnessed through the miracle of the market to maximize the potential of the economy. (Collier & Kay, 2020: 14).

Countless numbers of publications had preceded this one roughly offering the same message: utility is an empirically hollow and tautological concept, maximizing is not really how humans behave in daily practice, information is incomplete, precious, biased and potentially false, markets are always imperfect if not perversely organized into collusive monopolies or oligopolies, both production functions and innovation are ill-understood and treated like a black box and while description and explanation are disparaged as analytically inferior scientific activities, predictions are glorified but when produced by orthodox economists prove invariably deeply flawed if not downright harmful. For decades, the well-organized orthodox economists were able to withstand the pressure from more empirically inclined academics by presenting their exempting ceteris paribus clauses, by pointing at the beauty of conceptual parsimony and by vaunting the clarity of the numbers their calculations churned out to policymakers with eyes wide open and arms stretched out to receive them. But the 2008 financial crisis which once again struck the community of confident group-thinking economists like thunder in a clear sky, may well prove to have been a decisive turning point for their credibility. Since then, a distinct sense of *purpose* has emerged among a new generation of critical economists and business scholars. Although profit maximization as a behavioral driver among corporations is not completely washed away yet, its existence as the sole motive that makes shareholders tick and, directly responding to their demands, top managers as well, is jettisoned by a growing number of academics and practitioners. Instead business scholars such as Colin Mayer (2018) and Rebecca Henderson (2020) define the purpose of an organization as the key reason for its existence: what societal problem is it aimed at solving? Bicycle repair shops fix bicycles and perform well if their customers are happy with their service delivery, real estate agents do their job well when new owners of a dwelling have made the right choice and accountants may be satisfied with their work efforts if they have dug up most relevant information about another firm’s financial operations and fully applied their professional ethic to their report and final judgment, in their case even if the customer is less satisfied. In the end for organizations to fulfill this sense of purpose should prevail over making substantial levels of profit, even if the black figures are also in fine shape. This message is obviously attractive from a moral point of view and to some extent it is also true that many workers and employees are indeed driven by the content of their activities and quality of their efforts at least as much as by the financial gain they can provide to their superiors or shareholders. Heterodox economist Mariana Mazzucato (2021) frames it in a largely similar way: she sees as it the mission that an organization has formulated for itself aimed at creating broad societal value rather than narrowly circumscribed market value. This broader public value should then be brought in line exactly with Kate Raworth’s requirements for a doughnut economy.

The difficulty with this approach is at least twofold. The first complication is that organizational purpose cannot always be so unambiguously defined and is subject to change over time. The second is that many institutional and organizational incentives stimulate the maximization of profit and guide corporates towards behavior in which shareholder revenue is the highest good. As a result, corporate executives feel obliged to behave accordingly, squeeze as many funds out of their companies they lead as they possibly can and severely restrict the size of investment programs and the benefits for employees, suppliers and customers. They are also famous for rarely forgetting to look after their own interests. The question then becomes how profit-driven organizations can be turned into purpose-driven ones if profit is much more conveniently established than adherence to purpose and moreover strongly enforced by institutional and organizational rules in place. Practitioners and people with hands-on experience in business may easily discard this new purpose-oriented idealism as ‘naïve’. But not all is lost. Roche and Jakub (2017) in their ‘Completing capitalism’ argue that those companies that set up their supply chain in ways that maintain or even enhance human capital and natural capital among the participants in the supply chain are also the ones showing the best financial performance. In other words, environmentally and socially responsible behavior pays off handsomely in profit figures too, and they show this with a number of case studies. The good guys can never lose. In addition, proponents of new institutional winds such as Mazzucato (2015, 2021) also observe a lasting diminution and underestimation of the value of government and its importance in the creation (as opposed to the capturing) of public value. She proposes to make its position far more prominent again, both in terms of regulation and taxation imposed on corporations and to encourage them to turn their currently narrow focus on market value and shareholder value into one of broad public or societal value and stakeholder value. Government should not only regulate and tax the corporate sector, and especially the financial sector, far more strictly than it currently does to prevent it from undertaking operations that go against environmental and social inclusion. It should also take the lead in social and technological innovation again as it used to do before the Chicago School took control of economics and its political followers: government should trigger large complex projects again, undertake them in collaboration with corporate and civil society players and pay attention to receive the credits it deserves for its work in being at the origin of many pathbreaking technological innovations. In short, government should once again be a bold market-shaper rather than a timid market-fixer. It should retort accusations of government failure by pointing out that it is because of daring public funds rather than risk-avoiding private finance that society now has the disposal of high-quality health services, global internet and breath-taking imageries of spatial bodies lightyears away from planet Earth. Furthermore, where would the energy transition and other adjustments to limit climate change be without a steering role of public authorities alongside purpose-driven private companies? What would happen with the necessary education levels of large segments of the population if the private sector was in the driving seat? New-fangled economists and business scholars still see a role for Public–Private Partnerships, but rather than the private sector being the assertive side that is deemed efficient, successful and powerful with authorities merely picking the wining private bidders, it should be public players that take the lead: the days of public sector value creation and private sector value capturing are over; it is now time to curb the hoarding of the rentiers and give wider public values the boost they need to maintain social stability and ecological preservation. But are government and the corporate sector ready for this major switch?

## Reforming the varieties of capitalism towards more inclusiveness

Virtually all scholars in economics and business administration favoring the new ‘purpose paradigm’ evidence a strong preference for extensive (or at least enhanced) use of cooperatives in which means of production and finance are shared among the many, and for so-named stakeholder-oriented corporate structures. The latter refers to a style and structure of corporate management in which the various forces adducing resources to the company balance each other’s power out and each extract a substantial amount of benefit from it. Suppliers earlier in the value chain obtain higher, fair prices for what they deliver, environmental consequences of business operations are explicitly and/or compensated for, priced workers and employees at various level of hierarchy within the company enjoy rising salaries over time if increased revenues allow for it, sufficient funds are reserved for Research & Development and capacity growth, bank reserves are stuffed, customers enjoy discounts if the situation calls for it and are not systematically bombarded with sophisticated marketing campaigns to secure their mental dependency on products and services they do not truly need for their happiness, and shareholders still receive dividends but they no longer represent the main or only constituency with the primary right to financial and other benefits from the firm. Elsewhere this approach to corporate organization and management eventually finds its macro-societal counterpart in a stakeholder-oriented approach to broader socio-economic policies and institutions, as the Rhineland model (Albert, 1993), the Economics of Mutuality (Mayer & Roche, 2021) or the Coordinated Market Economy (CME) (Hall & Soskice, 2001). This stakeholder-oriented approach where various players in and around the organization negotiate over allocation and distribution of resources is normally contrasted with the shareholder-oriented approach, also known as the Anglo-Saxon model, the Economics of Extraction or the Liberal Market Economy (LME) in which financial capital clearly dominates all other types of capital. Hall and Soskice indicate that the economic performance of CMEs such as Germany and Japan, albeit both being of a very different kind, by and large are equal to those of LMEs such as the United States and the United Kingdom, but their levels of socio-economic inequality (GINI-index) are distinctly lower (Hall & Soskice, 2001). Other scholars, such as Lazonick and Shin (2020), Mazzucato (2018) and Streeck (2016) are more of the opinion that neither CMEs nor LMEs are sufficiently equipped to withstand the turbulent waves of ecological distress and socio-political instability humanity is faced with, but CMEs still tend to generate policy choices that are environmentally and socially more inclusive and therefore still come closer to the ideal of an inclusive capitalist system. In reality, there are obviously as many varieties of capitalism as there are nations in the world and it is the total constellation of how each of them establishes and changes the formal and informal institutions for the various key elements in its production and consumption processes: labor market, education system, R&D, welfare system, health system, corporate and investment legislation, intergovernmental relations, environmental regulation, taxation and many others, as well as the ways in which these components dynamically interconnect. There are theoretically as many possible varieties of capitalism as there combinations of all these features, all with somewhat different impact on environmental and social inclusion, all evolving in their own path-dependent ways addressing specific problems they face, how their public and private stakeholders address them in their own context and drawing selective lessons from other varieties of capitalism. Even though non-Western or unconventional economic systems such as Brazil, Russia, India and China are normally not included in the typology, their having in place or adoption of crucial principles for capitalism (see Sect. 2) also makes them specific varieties of capitalism, regardless of the their political system, nominal public ownership of land or divergent corporate management structures or styles (see also Lazonick, 1991; Hancke, 2009; Musacchio & Lazzarini, 2014; Clark, 2015). It would certainly go beyond the scope of this article to delve more deeply into a possible world classification of varieties (if such a typology could be convincingly produced at all), but a promising entry to examine how capitalism can be made more inclusive is by identifying various types of capital and how they are controlled and managed. Hodgson (2015) points out that depending on one’s theoretical perspective, capital can either be very broadly defined as ‘a relatively durable thing or attribute that leads to the satisfaction of wants’ or more narrowly as ‘the money value of tangible and intangible assets owned by the person or firm, which in principle can be used as collateral and serve to buy or hire resources to produce good or services for commodity exchange’ (2015: 184). He makes a choice for the latter and indicates that money capital and (physical) goods and services that can conveniently be converted into money may count as ‘real’ capital in the narrow sense of the word, while human, social and natural capital which cannot be normally be owned, bought and sold at market prices or measured in the aggregate, can only count as capital in the broader sense of the term.

However, to fulfill the task of boosting environmental and social inclusion in a capitalist system, it is inevitable also to value the capital contained in human knowledge, social networks and trust, and natural assets and let these be included in the calculation of human prosperity, even if they cannot be considered ‘real’ capital: they should somehow be conceptualized, measured and institutionalized as being of value too to be taken seriously in a *capital*ist society. In short, the whole approach to create and secure value and capture it in sustainable and equitable ways requires the incorporation of all types of capital in the prosperity equation: a complex yet urgent assignment. Setting an important step in that direction, Mayer (2018), Roche and Jakub (2017) and Mayer and Roche (2021) in their various books on prosperity, capitalism and mutuality systematically offer the same message: better business implies following ‘purpose’ as a yardstick for organizational behavior rather than profit. This implies mapping the full value chain of any product or service, establishing where environmental or social leakage takes place and insuring that at each node not only financial and physical, but also human, social and natural capital are at least kept at the same level and preferably enhanced. They do not only provide the concepts that make their perspective accessible to the broader government and business community, but also offer methods to operationalize their concepts, methods and metrics that allow users to measure their current performance and see where possible improvements lie. They have even developed an Oxford certification procedure that enables companies to have themselves inspected and advised in line with the ‘Economics of Mutuality’ procedures. Their vision is that corporations that do well on all relevant types of capital along the value chain are not only ethically better businesses, but that eventually this also pays off for them in handsome profits and satisfaction among all stakeholders. One may or may not follow the empirical implications of this logic, but skeptics can easily point out that this may be valid in certain niches of the markets or among specific firms, but that less willing corporations make superficial use of these metrics to greenwash their operations and that the laggards, the reluctant and the saboteurs will simply not undertake any action unless active public sector involvement forces them to do so and evasion of corporate responsibility is really no longer an option. The need for regulation, taxation, inspection and public campaigns automatically leads us to the role of government. Rebecca Henderson (2020), in spite of devoting most of her book to the need for corporate change, eventually clearly mentions how vital the role of government is in urging the private sector to undergo a profound transformation after which purpose will systematically have replaced profit as the main driver of organizational behavior. In sum, to realize inclusive capitalism leading authors in business administration must demand strong government both to play a leading role in making investments in a sustainable future and to undertake determined political and legal intervention among corporations that refuse to be a part of far-reaching changes required to keep the planet inhabitable for humans and to secure sufficient equality, solidarity and stability in our countries, cities and organizations. But are scholars in the field of politics and government preparing their students for this newly invigorated attitude?

## Implications for public policy as a discipline

The past 15 years or so have been particularly turbulent. The global financial crisis in 2008 and the COVID-19 pandemic have called into question many certitudes governments and industry thought they had about the nature of capitalism, democracy and rule of law. The operations of financial institutions and the public authorities supposed to monitor them have blatantly failed to serve the wider society they were believed to serve. The fragility of healthcare systems and political parties in dealing with crisis situations has become more apparent than many of us believed to be possible. Both crises could theoretically have been assumed to be blessings in disguise helping government, industry and civil society in most countries realize that a quantum leap in their policies vis-à-vis environmental and social inclusion in their capitalist systems was long overdue. But no such policy overhaul occurred: in spite of incidental pleas made here and there for societal transformation, public and private organizations as well as their citizens have been nearly universally eager to return to the original state before the outbreak of the financial crisis and corona epidemic as quickly as possible and avoid the complications of economic system change. Financial institutions were bailed out with funds provided by the tax-paying middle classes and returned to remunerating their shareholders and directors profusely as soon as normality had returned. Rather than ensuring that measures were taken to fundamentally alter modes of transport and bring the regulatory and taxation incentives for them more in line with the externalities they inflict on the natural environment, governments offered enormous financial support for their national airlines and endorsed mobility-hungry customers when they rushed to book cheap flights to their favorite holiday destinations as soon as travel restrictions had been lifted. On the face of it, then, it appears that the pessimists have it: it is only a matter of time until Kotkin’s (2020) era of inegalitarian neo-feudalism becomes a reality and Streeck’s (2016) prediction that post-capitalism will not be communism but a nondescript dark age clearly does not seem far off the mark.

But this does not have to be the end of the story. A host of forward-looking economists and business administration theorists have recently woken up to the new challenge and jettisoned many assumptions about their discipline which they had been taught and taken for granted for many years. They have begun to analyze the functioning of capitalism at the macro, meso and micro levels and infused it with a hue of empirical curiosity rather than self-confident model-making and enriched the world with the beginnings of what may well turn out to be the new *purpose paradigm*: corporate organizations do not and should not engage in profit maximization as the highest objective but return to the mission that brought them into being in the first place, which is good customer service through the delivery of quality goods and services that take the impact their creation has on the human and natural environment fully into account. Many leading theorists have made their coming out and engaged in both debunking old myths and proposing new metrics for making purpose the cornerstone of new forms of accounting and behavioral standards in the corporate world. This is a significant theoretical development of which the both the empirical merits and practical impact remains to be seen, but it definitely carries the stamp of a moral awakening that a more inclusive subspecies of capitalism sorely needs. What these theorists also do is clamor for a far more pro-active and daring government than they currently observe. If the coalition of the willing in the corporate world is to be effective in turning around the current shape of capitalism and bring it in line with that of a sustainable and egalitarian society, it requires pro-active, investing and market-shaping interventions from public organizations that level the playing field in view of these new conditions. Government as it functions now is simply too weak, too insecure and too permeated with neoliberal policy assumptions. Adherents of the purpose paradigm in economics and business know something urgently needs to change in politics, public policy, political theory and public policy theory. However, they are insufficiently aware of the internal operations within the public sector to tell *what* needs to change and *how* this can be changed. Public policy as an academic discipline is painfully lagging behind in its recognition that inclusive capitalism is more than managing policy networks, preparing packages of policy instruments, organizing open tenders for efficient services and analyzing discourses alone. Capitalism has always been a matter of money and installing power to collect and hoard it. An inclusive future needs more profound interventions of the public sector into the private sector to allow the good half of the latter to defeat the bad half of the latter. Without such regulatory and financial interventions based on solid legal and organizational foundations, it remains very probably that the bad corporate half will win. Capitalism is not a state, but a process and one in which public policy can and should play a role.

This contribution has consisted of an overview of recent heterodox developments in economic and business administration. It has identified the characteristics of a new purpose paradigm and sketched its challenges. It has presented the crucial elements of capitalism and inclusion and indicated that bringing these two concepts together is the only way to make our physical and social environments truly sustainable, but acknowledged that approaching this ideal requires value trade-offs that may look different around the world. In that sense, there are not one but many varieties of inclusive capitalism around the world. Finding an appropriate balance between pro-active public sector intervention to correct harmful private sector practices and corporate protection from arbitrary government infringement is not the same everywhere depends on specific institutional path-dependencies. But it is obvious that scholars in public policy should take heed of the abovementioned new theoretical developments in economics and business administration, come to a deeper and more critical understanding of capitalism and its institutions, their impact on processes of environmental and social exclusion and especially the role of government organizations in creating and recreating them. Charles Lindblom, undeniably an early protagonist in public policy, was known for (1) his awareness that policymaking is essentially a gradual and messy process of muddling through incomplete and ambiguous information and divergent interests through mutual adjustment (Lindblom & Woodhouse, 1992) and (2) his accurate and painful analysis of how pluralism in interest representation among various stakeholders was seriously impaired by the overwhelmingly influential lobby and impact of the corporate world on politicians and civil servants leading to systematic bias in the making of important political choices (Lindblom, 1990). Lindblom was an incrementalist when it came to how information is processed and an elitist in the way he looked at the distribution of power in political economies. He has often been vilified for producing two contradictory perspectives of politics and policy, but there is no real contradiction here. The world is indubitably complex and dynamic, but this does not exonerate policymakers and policy analysts from the duty always to bear the needs of the duped, underprivileged and the children in mind. Let Lindblom’s brilliant paradoxality be the guiding light for a thorough revision of public policy theory and practice. Capitalism can survive without it, but it is questionable whether an inclusive variety of it can.

## Data Availability

Not applicable.

## References

[CR1] Albert M (1993). Capitalism against capitalism.

[CR2] Aldred J (2019). Licence to be bad: How economics corrupted us.

[CR3] Anttiroiko AV, de Jong M (2020). The inclusive city: The theory and practice of creating shared prosperity.

[CR4] Bakan J (2004). The corporation: The pathological pursuit of profit and power.

[CR5] Bakan J (2020). The new corporation: How ‘good’ corporations are bad for democracy.

[CR6] Bell D (1976). The cultural contradictions of capitalism.

[CR7] Clark B (2015). The evolution of economic system: Varieties of capitalism in the global economy.

[CR8] Collier P (2018). The future of capitalism.

[CR9] Collier P, Kay J (2020). Greed is dead: Politics after individualism.

[CR10] Espino NA (2015). Building the inclusive city: Theory and practice for confronting urban segregation.

[CR11] Florida R (2003). The rise of the creative class: And how it is transforming work, leisure, community and every-day life.

[CR12] Florida R (2018). The new urban crisis: Gentrification, housing bubbles, growing inequality and what we can do about it.

[CR13] Fulcher J (2015). Capitalism: A very short introduction.

[CR14] Hall PA, Soskice D (2001). Varieties of capitalism: The institutional foundations of comparative advantage.

[CR15] Hambleton R (2014). Leading the inclusive city: Place-based innovation for a bounded planet.

[CR16] Hancke B (2009). Debating varieties of capitalism: A reader.

[CR17] Henderson R (2020). Reimagining capitalism in a world on fire.

[CR18] Hodgson GM (2015). Conceptualizing capitalism: Institutions, evolution, future.

[CR19] Kotkin J (2014). The new class conflict.

[CR20] Kotkin J (2020). The coming of neo-feudalism: A warning to the global middle class.

[CR21] Lazonick W (1991). Business organization and the myth of the market economy.

[CR22] Lazonick W, Shin JS (2020). Predatory value extraction: How the looting of the business corporation became the US norm and how sustainable prosperity can be restored.

[CR23] Lindblom CE (1990). Inquiry and change: The troubled attempt to understand and shape society.

[CR24] Lindblom CE, Woodhouse EJ (1992). The policy-making process.

[CR25] Mayer CP (2018). Prosperity: Better business makes the greater good.

[CR26] Mayer CP, Roche B (2021). Putting purpose into practice: The economics of mutuality.

[CR27] Mazzucato M (2015). The entrepreneurial state: Debunking public versus private sector myths.

[CR28] Mazzucato M (2018). The value of everything: Making and taking in the global economy.

[CR29] Mazzucato M (2021). Mission economy: A moonshot guide to changing capitalism.

[CR30] Musacchio A, Lazzarini SG (2014). Reinventing state capitalism: Leviathan in Business, Brazil and beyond.

[CR31] Piketty T (2017). Capital in the 21^st^ century.

[CR32] Pistor K (2019). The code of capital: How the law creates wealth and inequality.

[CR33] Raworth K (2017). Doughnut economics: Seven ways to think like a 21^st^ century economist.

[CR34] Roche B, Jakub J (2017). Completing capitalism: Heal business to heal the world.

[CR35] Steger MB, Roy RK (2021). Neoliberalism: A very short introduction.

[CR36] Streeck W (2016). How will capitalism end?: Essays on a failing system.

[CR37] Williamson OE (1990). The economic institutions of capitalism.

